# The reporting standards of randomised controlled trials in leading medical journals between 2019 and 2020: a systematic review

**DOI:** 10.1007/s11845-022-02955-6

**Published:** 2022-03-03

**Authors:** Mairead McErlean, Jack Samways, Peter J. Godolphin, Yang Chen

**Affiliations:** 1 UCLPartners, London, UK; 2 Cardiology Department, London North West University Healthcare NHS Trust, London, UK; 3 MRC Clinical Trials Unit at University College London, Institute of Clinical Trials and Methodology, University College London, London, UK; 4 Institute of Health Informatics, University College London, 222 Euston Road, London, NW1 2DA UK

**Keywords:** CONSORT, Randomised controlled trial (RCT), Reporting guidelines

## Abstract

Randomised controlled trials (RCTs) are the gold standard study design used to evaluate the safety and effectiveness of healthcare interventions. The reporting quality of RCTs is of fundamental importance for readers to appropriately analyse and understand the design and results of studies which are often labelled as practice changing papers. The aim of this article is to assess the reporting standards of a representative sample of randomised controlled trials (RCTs) published between 2019 and 2020 in four of the highest impact factor general medical journals. A systematic review of the electronic database Medline was conducted. Eligible RCTs included those published in the *New England Journal of Medicine*, *Lancet*, *Journal of the American Medical Association*, and *British Medical Journal* between January 1, 2019, and June 9, 2020. The study protocol was registered on medRxiv (https://doi.org/10.1101/2020.07.06.20147074). Of a total eligible sample of 497 studies, 50 full-text RCTs were reviewed against the CONSORT 2010 statement and relevant extensions where necessary. The mean adherence to the CONSORT checklist was 90% (SD 9%). There were specific items on the CONSORT checklist which had recurring suboptimal adherence, including in title (item 1a, 70% adherence), randomisation (items 9 and 10, 56% and 30% adherence) and outcomes and estimation (item 17b, 62% adherence). Amongst a sample of RCTs published in four of the highest impact factor general medical journals, there was good overall adherence to the CONSORT 2010 statement. However there remains significant room for improvement in areas such as description of allocation concealment and implementation of randomisation.

**Supplementary Information:**

The online version contains supplementary material available at 10.1007/s11845-022-02955-6.

## Introduction

Randomised controlled trials (RCTs) are the gold standard study design used to evaluate the safety and effectiveness of healthcare interventions [1]. When published in academic journals, many years of work, often at significant financial cost, may be summarised in fewer than 5000 words. It is therefore imperative that the reporting quality of an RCT is transparent and clear, allowing readers to appropriately analyse and understand the study design and results that may change clinical practice. In an attempt to improve trial reporting, the Consolidated Standards of Reporting Trials (CONSORT) statement was developed in 1996 [2]. Since its inception, there have been significant updates in 2001 and 2010 [3, 4]. The current iteration consists of a 25-item checklist for correct trial reporting.

Previous studies have shown poor reporting standards in RCTs, particularly in areas concerning trial methodology [5–8]. More recently, a 2016 systematic review of reporting standards of RCTs within Cardiology reinforced this point—adherence to CONSORT items was only 63.8% (SD 18.1%) [9].

This study aims to assess the reporting standard of a representative sample of RCTs published between 2019 and 2020 four of the highest impact general medical journals [9]. Given the dramatic changes that the COVID-19 pandemic has brought to academia, including greater use of pre-prints and scrutiny of the data veracity of studies, the importance of robust reporting of RCTs is more salient that ever.

## Methods

The protocol for this systematic review was registered before data extraction, as a preprint on the medRxiv database https://doi.org/10.1101/2020.07.06.20147074, following an initial attempt to register on PROSPERO. This was declined on the grounds of not fulfilling the scope of PROSPERO, despite similar works being registered [10]. This manuscript has been prepared according to the updated 2020 guidelines issued by the PRISMA group [11]. A checklist is available in the Supplemental Appendices along with a list of protocol deviations and justifications.

### Eligibility criteria

Studies were eligible for inclusion if they (1) described the primary results of a randomised trial; (2) were published in the *New England Journal of Medicine*, the *Lancet*, the *Journal of the American Medical Association* or the *British Medical Journal* articles; and (3) were English language publications.

### Study identification

We performed a comprehensive search of the terms ‘New England Journal of Medicine’, ‘Lancet’, ‘Journal of the American Medical Association’ and ‘British Medical Journal’ on MEDLINE only, given that these target journals are all MEDLINE indexed. To identify RCTs for inclusion, the Cochrane Highly Sensitive Search Strategies for identifying randomised trials filter was used [12]. A time filter was applied to obtain results from January 1, 2019, to June 9, 2020 (the date of the search execution).

### Study selection and data extraction

After removal of duplicates and clearly irrelevant records, two independent reviewers (MM, JS) screened the titles and abstracts of the search results. The full texts of the remaining results were individually assessed by both reviewers for inclusion with arbitration by a third author if necessary (YC).

Data from eligible studies was extracted from study reports independently by three reviewers (MM, JS and PG) including the general characteristics of the RCTs.

### Adherence to reporting standards

A random sample of 50 papers was selected to score against the CONSORT checklist using a random number list generated by one reviewer (PG). The software package Stata SE version 16.0 (StataCorp, College Station, TX, USA) was used to generate the random number list. Papers were scored independently by three authors (MM, JS and PG) against the 25-item 2010 CONSORT statement.

Each item was given an equal weighting—12 items were divided into A and B parts giving a total of 37 points scored per paper. Each item was subdivided as outlined in the CONSORT statement [4]. Any differences in scores were resolved through consensus. The CONSORT Extension statement was used for RCTs that included designs other than a parallel 2-arm comparison [13–15]. Two papers from each scorer were selected at random and audited independently by the corresponding author (YC). If auditing revealed significant discrepancies, a re-evaluation of the original scoring was triggered.

### Risk of bias and data synthesis

We did not conduct a risk of bias assessment nor a quantitative synthesis.

## Results

### Identified and eligible studies

**Fig. 1:**
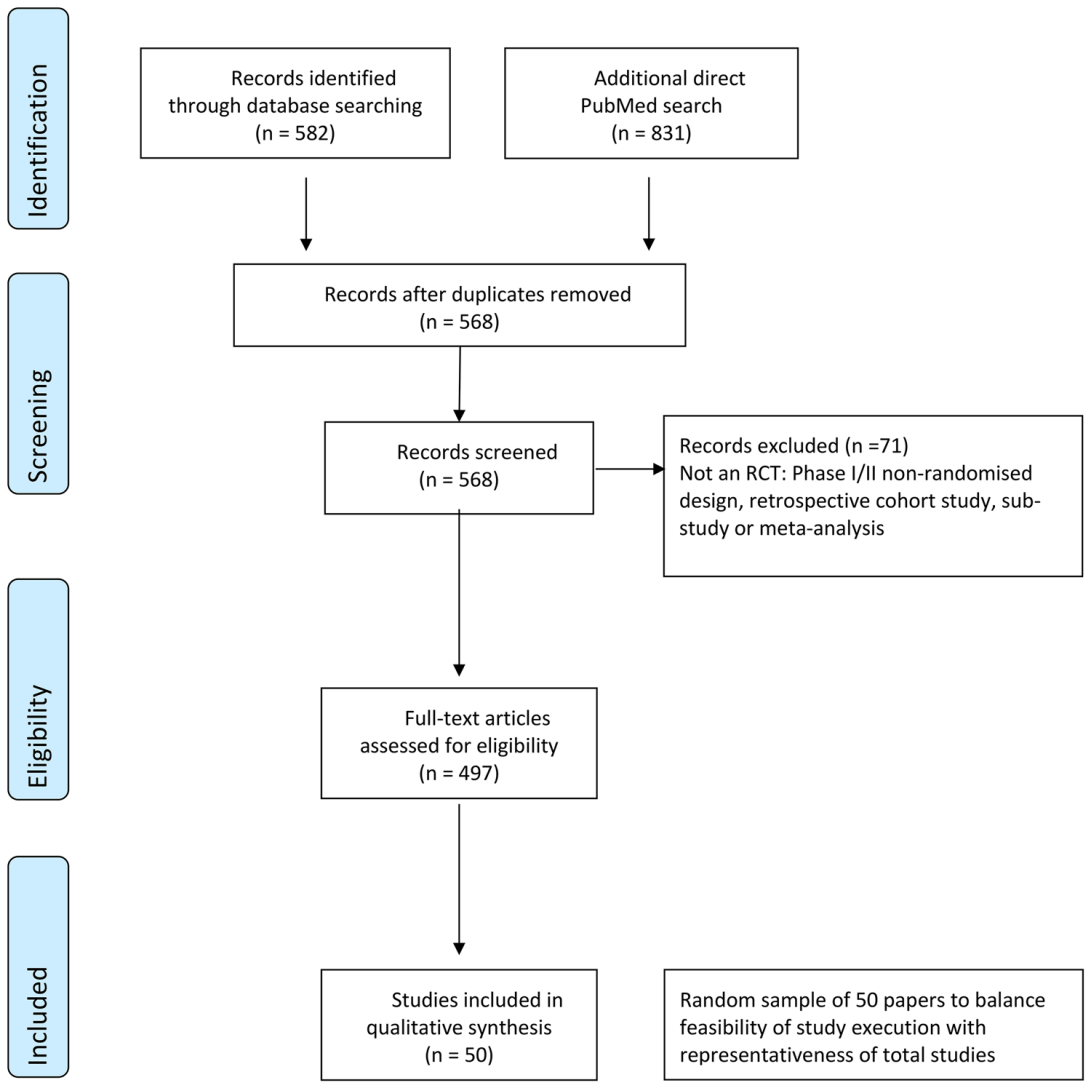
PRISMA Flow of study records

A total of 1,413 records were retrieved by electronic searches, last updated on June 9, 2020 (see Fig. 1). After removal of duplicates, 568 full texts were assessed of which 71 were excluded. A final list of 50 papers was chosen at random for full analysis against the CONSORT statement.

Of the 50 RCTs selected for full CONSORT scoring, their general characteristics are summarised in Table 1. Their representativeness compared to the 497 eligible RCTs is described, along with a full table of characteristics of all eligible studies, in the supplementary materials. The overall adherence to CONSORT items was high—across all the studies, the average adherence was 90% (SD 9%), and only 4 RCTs (8%) scored less than 75% adherence to relevant CONSORT 2010 items (Table 2). Compliance was variable between different CONSORT 2010 items. This is shown in Table 3 and was poorest for items relating to title, item 1a (35/50, 70%); allocation concealment, item 9 (28/50, 56%); implementation, item 10 (15/50, 30%); and outcomes and estimation, item 17b (21/34, 62%).

**Table 1:** Summary characteristics of RCTs included for qualitative analysis

**Journal**	**Specialty area**	**Design**
BMJ (*n* = 3)^*^	Oncology (*n* = 6)	Parallel group 2-arm (*n* = 40)
JAMA (*n* = 14)	Cardiovascular (*n* = 13)	Parallel group > 2 arms (*n* = 3)
Lancet (*n* = 16)	Infectious disease (*n* = 8)	Cluster (*n* = 4)
NEJM (*n* = 17)	Inflammatory (*n* = 6)	Factorial (*n* = 3)
	Surgical (*n* = 8)	
	Other (*n* = 9)	

Characteristics of 50 included studies

*BMJ* British Medical Journal, *JAMA* Journal of American Medical Association, *NEJM* New England Journal of Medicine

^*^Representative of overall sample of 497, where only 20 RCTs were from BMJ/115 from JAMA/151 from Lancet and 211 from NEJM

**Table 2:** List of selected studies and their adherence to the CONSORT 2010 Statement or relevant extension and the % adherence when assessed against relevant items

**Author**	**Journal**	**RCT design**	**Number of adhered CONSORT items**	**Number of relevant CONSORT items**	**Overall CONSORT adherence**
Al Batran et al	Lancet	Parallel group 2-arm	32	33	97%
Bernitz et al	Lancet	Cluster randomised parallel group 2-arm	30	33	91%
Burtness et al	Lancet	Parallel group 3-arm	30	34	88%
Campbell et al	JAMA	Parallel group 2-arm	30	33	91%
Claassens et al	NEJM	Parallel group 2-arm	30	35	86%
Cohen et al	Lancet	Parallel group 2-arm	34	34	100%
Diener et al	NEJM	Parallel group 2-arm	30	34	88%
Fisher et al	JAMA	Parallel group 2-arm	32	34	94%
Futier et al	JAMA	parallel group 2-arm	32	35	91%
Gimbel et al	Lancet	Parallel group 2-arm	32	34	94%
Gonzalez-Martin et al	NEJM	Parallel group 2-arm	31	37	84%
Hajek et al	NEJM	Parallel group 2-arm	31	33	94%
Hanley et al	Lancet	Parallel group 2-arm	31	34	91%
Hausenloy et al	Lancet	Parallel group 2-arm	32	34	94%
Havlir et al	NEJM	Cluster randomised parallel group 2-arm	23	34	68%
Huang et al	NEJM	Parallel group 2-arm	29	33	88%
Issa et al	JAMA	Parallel group 2-arm	34	34	100%
Keene et al	BMJ	Parallel group 2-arm	32	33	97%
Khorana et al	NEJM	Parallel group 2-arm	26	32	81%
Kim et al	JAMA	Parallel group 2-arm	30	32	94%
Kortekangas et al	BMJ	Noninferiority Parallel group 3-arm	32	32	100%
Kroon et al	Lancet	Parallel group 2-arm	34	34	100%
Lemkes et al	NEJM	Parallel group 2-arm	23	30	77%
Liu-Ambrose et al	JAMA	Parallel group 2-arm	33	33	100%
Makkar et al	NEJM	Parallel group 2-arm	22	32	69%
Manson et al	NEJM	2 × 2 Factorial	19	33	58%
Masa et al	Lancet	Parallel group 2-arm	30	33	91%
McCann et al	Lancet	Parallel group 2-arm	33	33	100%
Mehanna et al	Lancet	Parallel group 2-arm	29	30	97%
Milstone et al	JAMA	Parallel group 2-arm	34	35	97%
Nagel et al	NEJM	Noninferiority Parallel group 2-arm	25	29	86%
Parsons et al	JAMA	Parallel group 2-arm	28	30	93%
Pittock et al	NEJM	Parallel group 2-arm	32	36	89%
Rosenstock et al	JAMA	Parallel group 2-arm	29	33	88%
Sands et al	NEJM	Parallel group 2-arm	25	34	74%
Schupke et al	NEJM	Parallel group 2-arm	30	33	91%
Shehabi et al	NEJM	Parallel group 2-arm	29	33	88%
Sheppard et al	JAMA	Parallel group 2-arm	32	33	97%
Skjerven et al	Lancet	2 × 2 factorial, cluster randomised	32	33	97%
Spahn et al	Lancet	Parallel group 2-arm	29	33	88%
Staedke et al	Lancet	Cluster randomised 2-arm	32	34	94%
Tang et al	BMJ	Parallel group 2-arm	33	33	100%
Taylor et al	Lancet	Parallel group 3-arm	32	33	97%
van Kempen et al	NEJM	Parallel group 2-arm	28	33	85%
von Dach et al	JAMA	Parallel 3-arm	31	33	94%
Walsh et al	NEJM	2 × 2 factorial	30	34	88%
Wolf et al	JAMA	× 2 Parallel group 2-arm	29	33	88%
Writing Committee for the PROBESE Collaborative Group	JAMA	× 2 Parallel group 2-arm	33	35	94%
Young et al	JAMA	Cluster crossover	31	34	91%
Younossi et al	Lancet	× 2 Parallel group 2-arm	32	34	94%

**Table 3:** CONSORT 2010 adherence by individual item. For items 3b, 6b, 7b, 11a, 11b, 12b, 17b and 18, these were not applicable for many of the included sample for analysis

**Section/topic**	**Item no**	**Checklist item**	**Adherence**
Title and abstract	1a	Identification as a randomised trial in the title	35/50 (70%)
	1b	Structured summary of trial design, methods, results and conclusions (for specific guidance see CONSORT for abstracts)	50/50 (100%)
Background and objectives	2a	Scientific background and explanation of rationale	47/50 (94%)
	2b	Specific objectives or hypotheses	44/50 (88%)
Trial design	3a	Description of trial design (such as parallel, factorial) including allocation ratio	48/50 (96%)
	3b	Important changes to methods after trial commencement (such as eligibility criteria), with reasons	7/8 (88%)
Participants	4a	Eligibility criteria for participants	49/50 (98%)
	4b	Settings and locations where the data were collected	44/50 (88%)
Interventions	5	The interventions for each group with sufficient details to allow replication, including how and when they were actually administered	50/50 (100%)
Outcomes	6a	Completely defined pre-specified primary and secondary outcome measures, including how and when they were assessed	48/50 (96%)
	6b	Any changes to trial outcomes after the trial commenced, with reasons	6/6 (100%)
Sample size	7a	How sample size was determined	45/50 (90%)
	7b	When applicable, explanation of any interim analyses and stopping guidelines	21/21 (100%)
Randomisation:			
Sequence generation	8a	Method used to generate the random allocation sequence	43/50 (86%)
	8b	Type of randomisation; details of any restriction (such as blocking and block size)	47/50 (94%)
Allocation concealment mechanism	9	Mechanism used to implement the random allocation sequence (such as sequentially numbered containers), describing any steps taken to conceal the sequence until interventions were assigned	28/50 (56%)
Implementation	10	Who generated the random allocation sequence, who enrolled participants and who assigned participants to interventions	15/50 (30%)
Blinding	11a	If done, who was blinded after assignment to interventions (for example, participants, care providers, those assessing outcomes) and how	41/43 (95%)
	11b	If relevant, description of the similarity of interventions	8/11 (73%)
Statistical methods	12a	Statistical methods used to compare groups for primary and secondary outcomes	49/50 (98%)
	12b	Methods for additional analyses, such as subgroup analyses and adjusted analyses	43/45 (96%)
Participant flow (a diagram is strongly recommended)	13a	For each group, the numbers of participants who were randomly assigned, received intended treatment and were analysed for the primary outcome	46/50 (92%)
	13b	For each group, losses and exclusions after randomisation, together with reasons	47/50 (94%)
Recruitment	14a	Dates defining the periods of recruitment and follow-up	48/50 (96%)
	14b	Why the trial ended or was stopped	48/50 (96%)
Baseline data	15	A table showing baseline demographic and clinical characteristics for each group	47/50 (94%)
Numbers analysed	16	For each group, number of participants (denominator) included in each analysis and whether the analysis was by original assigned groups	48/50 (96%)
Outcomes and estimation	17a	For each primary and secondary outcome, results for each group and the estimated effect size and its precision (such as 95% confidence interval)	47/50 (94%)
	17b	For binary outcomes, presentation of both absolute and relative effect sizes is recommended	21/34 (62%)
Ancillary analyses	18	Results of any other analyses performed, including subgroup analyses and adjusted analyses, distinguishing pre-specified from exploratory	46/46 (100%)
Harms	19	All important harms or unintended effects in each group (for specific guidance see CONSORT for harms)	47/50 (94%)
Limitations	20	Trial limitations, addressing sources of potential bias, imprecision and, if relevant, multiplicity of analyses	47/50 (94%)
Generalisability	21	Generalisability (external validity, applicability) of the trial findings	44/50 (88%)
Interpretation	22	Interpretation consistent with results, balancing benefits and harms and considering other relevant evidence	50/50 (100%)
**Other information**			
Registration	23	Registration number and name of trial registry	50/50 (100%)
Protocol	24	Where the full trial protocol can be accessed, if available	50/50 (100%)
Funding	25	Sources of funding and other support (such as supply of drugs), role of funders	50/50 (100%)

## Discussion

Our study has three principal findings. Firstly, the general reporting standard of RCTs in high impact medical journals in 2019–2020 is strong. Nine papers had either full or all but one item fully adherent to the CONSORT 2010 guidance. Second, there were common and important areas where performance was suboptimal—namely in allocation concealment and implementation of randomisation. Third, there was a significant number of cluster and factorial study designs—14% of the studies analysed were scored using their relevant CONSORT extension document.

Our findings demonstrate a marked improvement from previous systematic reviews [7, 8] which admittedly included RCTs from a more heterogeneous group of journals. More broadly, whilst the adoption and use of reporting guidelines are prominent in RCTs, there remains work to be done across other study designs and areas of medicine, notably highlighted in a recent paper examining high impact rehabilitation journals [16]. Other groups have considered additional extensions to CONSORT—in particular a more detailed description of the interventions used in RCTs [17]. How this becomes blended together with other initiatives to achieve a consistently high standard of RCT reporting remains a challenge for future work.

In current RCT reporting published within journals that represent the pinnacle of academia, areas of concern remain. The reporting of important steps in the randomisation process such as allocation concealment and implementation are areas of weakness. Whilst editors are confined by limited journal space, there may be a role for 100–200 words of protected text to allow for adequate description of the main strength of an RCT design—the component of randomisation. As an editorial described at the time of release of CONSORT, if ‘the whole of medicine depends on the transparent reporting of clinical trials’ [1], then the cost of an extra couple of paragraphs or a clearly signposted and structured appendix to find detailed descriptions of the randomisation process should be prioritised, given that robust methodology is held in as high regard as the result of the trial itself.

The reporting of some basic CONSORT items remains consistently poor across time; for instance, omission of the word ‘randomised’ in the title was noted in nearly half of CONSORT abstracts of the same medical journals in a 2012 analysis [18]. There is no clear explanation for why this remains the case though the definition of quality of reporting is not simply limited to CONSORT checklist adherence. Indeed one could argue that for journals such as the NEJM, article titles do not require explicit conformity given the entry criteria for publishing original research on their platform necessitates robust methodological design.

Lastly, the word count limits imposed by the journals vary considerably—ranging from 2700 words in the NEJM to an unrestricted upper end in the BMJ (see supplementary materials). We noted that whilst many of the sections are interchangeable across the different journal platforms, there were advantages to the more liberal constraints of the BMJ, particularly with regard to mandatory reporting of patient and public involvement, and thus focusing readers’ attention in thinking about the value of the study that they are reading with reference to the views and input of patients who may benefit from such results. Whilst a counter-argument to restricted space is that there is almost always co-publication of supplementary material which can contain helpful additional information to the reader, what remains unknown is the number of times such material is ever downloaded and read.

### Limitations

Our findings must be considered in the light of several limitations. First, there is inherent subjectivity to the way that adherence to CONSORT items are judged. Although we used independent reviewers and adjudication to reach consensus, there may nevertheless remain small variation in findings if results were to be replicated. Second, we chose only 50 papers to be scored—this was to balance against feasibility and comprehensiveness, and our sample size is similar to existing reviews which have examined reporting standards [7, 8]. Random sampling represented the most robust method to select papers from our initial eligible list, though this may not capture the extent to which CONSORT scores may vary depending on different study designs or subject areas. If a larger sample was assessed, then possible associations could be uncovered. Of note, there is also a significant difference between the number of articles contributed by each journal—although this is broadly representative of the full 497 studies identified, the relative contribution of each journal is unequal and should temper overall conclusions.

Lastly, the choice of restriction to four of the highest impact general medical journals has substantially limited the number of RCTs that were possible to be analysed, of which many have a wide readership within specialty subject areas—for example, the European Heart Journal. Our contention is that by focusing on the journals with the largest academic circulation and readership, the assumption is that the reporting standards of the published RCTs are likely to be amongst the best within the research community. RCTs in other journals will likely require just as much if not more focus on ensuring that their reporting quality remains as close to what is advocated by the CONSORT group as possible—a standard that has garnered almost universal support amongst quality medical journals.

## Conclusion

Amongst RCTs published between 2019 and 2020 within four of the highest impact factor general medical journals, there is strong adherence to the CONSORT 2010 statement. Specific components still have room for improvement, with allocation concealment and implementation of the randomisation process representing areas for particular focus.

## Supplementary Information

Below is the link to the electronic supplementary material.

Supplementary file1 (DOCX 19 KB)

Supplementary file2 (PDF 697 KB)

## Data Availability

See supplement.
