# Correction to: The reporting standards of randomised controlled trials in leading medical journals between 2019 and 2020: a systematic review

**DOI:** 10.1007/s11845-022-02969-0

**Published:** 2022-03-17

**Authors:** Mairead McErlean, Jack Samways, Peter J. Godolphin, Yang Chen

**Affiliations:** 1 UCLPartners, London, UK; 2 Cardiology Department, London North West University, Healthcare NHS Trust, London, UK; 3 MRC Clinical Trials Unit at University College London, Institute of Clinical Trials and Methodology, University, College London, London, UK; 4 Institute of Health Informatics, University College London, 222 Euston Road, London NW1 2DA, UK

## Correction to: Irish Journal of Medical Science (2022) https://doi.org/10.1007/s11845-022-02955-6

The original article contains an error. See below;

In the abstract, it should read ‘Of a total eligible sample of **497** studies,…’.

In the results section, it should read ‘After removal of duplicates, 568 full texts were assessed of which **71** were excluded….’.

The original article has been corrected.

