# Supplementary material for: The reporting standards of randomised controlled trials in leading medical journals between 2019 and 2020: a systematic review
Source: Ir J Med Sci. 2022 Mar 3;192(1):73–80. doi: 10.1007/s11845-022-02955-6 (PMC8890950; doi:10.1007/s11845-022-02955-6)
Supplement: Supplementary file 1 — Supplementary file1 (DOCX 19 KB) [file 11845_2022_2955_MOESM1_ESM.docx]

### APPENDIX 1 – SEARCH STRATEGY

**Medline (OvidSP)**

| 1. | JAMA.mp. or journal of the american medical association.jn. [mp=title, abstract, heading word, drug trade name, original title, device manufacturer, drug manufacturer, device trade name, keyword, floating subheading word, candidate term word] |  |  |  |
| --- | --- | --- | --- | --- |
| 2. | bmj.mp. or british medical journal.jn. [mp=title, abstract, heading word, drug trade name, original title, device manufacturer, drug manufacturer, device trade name, keyword, floating subheading word, candidate term word] |  |  |  |
| 3. | lancet.jn. |  |  |  |
| 4. | nejm.mp. or new england journal of medicine.jn. [mp=title, abstract, heading word, drug trade name, original title, device manufacturer, drug manufacturer, device trade name, keyword, floating subheading word, candidate term word] |  |  |  |
| 5. | 1 or 2 or 3 or 4 |  |  |  |
| 6. | ("clinical trial" or "clinical trial, phase i" or "clinical trial, phase ii" or clinical trial, phase iii or clinical trial, phase iv or controlled clinical trial or "multicenter study" or "randomized controlled trial").pt. or double-blind method/ or clinical trials as topic/ or clinical trials, phase i as topic/ or clinical trials, phase ii as topic/ or clinical trials, phase iii as topic/ or clinical trials, phase iv as topic/ or controlled clinical trials as topic/ or randomized controlled trials as topic/ or early termination of clinical trials as topic/ or multicenter studies as topic/ or ((randomi?ed adj7 trial*) or (controlled adj3 trial*) or (clinical adj2 trial*) or ((single or doubl* or tripl* or treb*) and (blind* or mask*))).ti,ab,kw. or ("4 arm" or "four arm").ti,ab,kw. |  |  |  |
| 7. | 5 and 6 |  |  |  |
| 8. | limit 7 to yr="2019 -Current" |  |  |  |

**🡪 582 records on 9 June 2020**

The same search strategy was used on PubMed directly and this generated 831 records when searched on 16 June 2020. Both were then combined to ensure a comprehensive list was generated.

### APPENDIX 2 – PROTOCOL DEVIATIONS

**Scope of review**

### The original protocol sought to answer the following questions:

### To systematically identify RCTs published in the four leading medical journals between January 1^st^ 2019 – May 31^st^ 2020

- To assess the quality of reporting of such RCTs using the CONSORT 2010 statement
- To identify any association with medical specialty or size or type of RCT and the rate of adherence to the CONSORT 2010 statement.

The following changes, along with justifications, were made to the protocol:

- To include only a random sample of 50 RCTs for full analysis of their adherence to the CONSORT 2010 statement. This decision was taken in light of the volume of RCTs that were returned that fulfilled the search eligibility criteria, ensuring a timely and feasible analysis of a representative sample of studies. The number of RCTs analysed are of a comparable size to previously published work in the area [see references].
- No associations with regards to medical specialty, size or type of RCT and adherence to CONSORT 2010 statement were tested, owing to the reduced sample size that was assessed

**APPENDIX 3 - Minimisation and resolution of differences in CONSORT scoring**

The following process was followed by the study group when undertaking analysis of CONSORT scoring:

Step 1 – calibration. Use of existing published work & group discussion to generate ‘thresholds’ for whether a paper would be given the mark or not for individual items on the CONSORT 2010 checklist. Many items were clear-cut e.g. Item 1a. Others benefited from this calibration e.g. Item 7a and determining that the threshold for ‘scoring’ would be that it would have to feature in the main manuscript.

Step 2 – conflict resolution. Significant discrepancies were considered as greater than two differences for any individual paper between reviewers and the auditor.

**APPENDIX 4 - Summary of formatting guidance of journals:**

NEJM:

https://www.nejm.org/author-center/article-types

- Up to 2700 words

Lancet:

https://marlin-prod.literatumonline.com/pb-assets/Lancet/authors/tl-info-for-authors.pdf

- Up to 4500 words.

JAMA:

https://jamanetwork.com/journals/jama/pages/instructions-for-authors

- Up to 3000 words.

BMJ:

https://www.bmj.com/about-bmj/resources-authors/article-types

- no fixed word limit

**APPENDIX 5 – Distribution of RCTs across the journals**

| **Journal** | Included studies (n=50) | All eligible studies (n=497) |
| --- | --- | --- |
| BMJ | 3 (6%) | 20 (4%) |
| JAMA | 14 (28%) | 115 (23%) |
| Lancet | 16 (32%) | 151 (30%) |
| NEJM | 17 (34%) | 211 (43%) |

For the full list of individual studies, please see attached supplementary file
